# Integrated health-environment-economy approach in the Brazilian Amazon: mapping production landscape units

**DOI:** 10.1590/0102-311XEN066424

**Published:** 2025-03-24

**Authors:** Anielli Rosane de Souza, Maria Isabel Sobral Escada, Ana Paula Dal’Asta, Marcus Vinicius Gonçalves da Silva, Danilo Araújo Fernandes, Antonio Miguel Vieira Monteiro

**Affiliations:** 1 Instituto Nacional de Pesquisas Espaciais, São José dos Campos, Brasil.; 2 Universidade Federal de Lavras, Lavras, Brasil.; 3 Universidade Federal do Pará, Belém, Brasil.

**Keywords:** Remote Sensing Technology, Amazonian Ecosystem, Rural Economy, Public Health Surveillance, Classification, Tecnología de Sensores Remotos, Ecosistema Amazónico, Economia Rural, Vigilancia em Salud Pública, Clasificación

## Abstract

The agrarian economy, with its social agents and technical systems, mobilizes the elements that generate transformations in the social and natural landscapes in the Brazilian Amazon. Choices for regional development lead to sustainability or unsustainability of the forest ecosystem and its social landscape, while not including health in this debate. We argue that an analytical framework for integrated health-environment-economy approaches needs a territorial representation for the landscapes associated with the ways of living and producing in Amazonian agriculture: the production landscape units (PLU). In this article, we explore machine learning techniques, in the field of supervised classification, with methods based on decision trees, to identify and map the PLU. A case study is developed for the municipalities of Mocajuba and Cametá, in the Baixo Tocantins region, in the State of Pará, for 2021. We describe how to identify and map the PLU in an intra-municipal spatial unit of reference and how to associate them with the types of rural techno-productive trajectories or technological trajectories (TTs) found in the regional agrarian economy. We promote an initial discussion on the use of PLU in the structuring of integrated approaches in health. This article contributes to align debates on strategies for economic development with health promotion in the Brazilian Amazon.

## Abstract (es)

La economía agraria, con sus agentes sociales y sistemas técnicos, moviliza los elementos que generan transformaciones en los paisajes social y forestal en la Amazonia brasileña. Las opciones para el desarrollo regional conducen a la sostenibilidad o insostenibilidad del ecosistema forestal y de su paisaje social. En este debate se descuida la salud. Sostenemos que un marco analítico para enfoques integrados salud-ambiente-economía requiere una representación territorial de los paisajes asociados con las formas de vivir y producir en la agricultura amazónica, las unidades de paisaje productivo (PLU, por su sigla en inglés). En este artículo exploramos técnicas de aprendizaje automático, en el campo de la clasificación supervisada, con métodos basados en árboles de decisión, para identificar y mapear PLU. Se desarrolla un estudio de caso para los municipios de Mocajuba y Cametá, en la región del Baixo Tocantins, en el Estado de Pará, para el año 2021. Describimos cómo identificar y mapear las PLU en una unidad espacial de referencia intramunicipal y cómo asociarlos con los tipos de trayectorias tecnoproductivas rurales o trayectorias tecnológicas (TT) presentes en la economía agraria regional. Promovemos una discusión inicial sobre el uso de las PLU en la estructuración de enfoques integrados de salud. Este artículo contribuye a alinear los debates sobre estrategias de desarrollo económico con la promoción de la salud en la Amazonia brasileña.

## Introduction

Analytical approaches to health-disease processes in the Brazilian Amazon should offer a framework that integrates the triad of health, environment, and economy. The agrarian economy ^1^, with its diverse agents and technical systems, is the main factor that mobilizes the elements that drive transformations in the social and natural landscapes of the Amazon. Such transformations create anthropogenic disturbances in the forest ecosystem and affect its structural and functional integrity ^2^, changing land use and cover patterns, the role of socio-spatial units, and their arrangement in the contemporary Amazonian urban environment ^3^. They also change the distribution of income, goods, and services associated with the chains involved in regional and local economies. In this perspective, these technical systems of production are directly related to the choice of a regional development path that can lead to the sustainability or unsustainability of the forest and social landscape, interconnected to the history of occupation of the Brazilian Amazon ^4^ ^,^ ^5^. In this context, the health dimension is scarcely addressed in these debates. In order to promote this analytical structure, a new territorial representation is necessary for the landscapes of the biome, organized as production landscape units (PLU), as we will argue throughout this article. The PLU are units associated with the modes of production operated by the rural techno-productive systems, present in the agrarian economy.

The PLU is conceived as a representation for the state of forest landscapes modified by technical systems associated with rural production. In the Amazon context, the PLU encompass a variety of ecosystems and and environments that are interspersed and overlap as a result of the interactions between production agents, such as farmers, extractivists, cattle ranchers and local communities, and the components of the landscape, such as rivers, forests, roads and cities. These elements are organized in varied and non-uniform spatial arrangements, reflecting the diversity and complexity of social interactions, economic and environmental interactions ^6^ ^,^ ^7^ ^,^ ^8^. Once identified and mapped, the PLU can be used as a proxy, that is, as a surrogate indicator variable, for the types of technical systems associated with rural production in the area under analysis. The rural techno-productive trajectories or technological trajectories (TTs) are analytical categories introduced by Costa ^9^ ^,^ ^10^ for the analysis of agrarian economics. They represent patterns of technical and productive solutions that are constituted as specific trajectories, adopted by various rural (either peasant or capitalist) production units.

The patterns observed in the modified landscapes are influenced by the ways of living and modes of production, associated with the choices of production models of peasant and capitalist agents. The analysis of specific spatial patterns, observed in modified landscapes, makes it possible to associate them with the corresponding PLU and TTs. As the PLU represent landscapes constantly shaped by the technical systems of production, these spatial patterns − typically present in the living spaces of these agents − can be identified and measured via satellite images.

Codeço et al. ^11^ highlight the importance of integrated approaches considering economy, environment, and health-disease processes to (re)formulate health surveillance strategies. The article ^11^ offers a framework to discuss the economic, environmental, and health dimensions at the municipal level.

The economic dimension is analyzed based on the analytical category defined by Costa ^4^ ^,^ ^9^ as a descriptor of the agrarian economy in the Amazon, the TTs. Costa ^10^, using data from the agricultural censuses (1995, 2006, and 2017) in a methodology based on statistical regressions, principal components, and factor analysis, shows the presence of six types of TTs in the municipalities of the Brazilian Amazon. Codeço et al. ^11^ associates the dominant TT in the municipal agrarian economy with environmental and epidemiological indicators from a database organized and harmonized at the municipal level ^12^.

The methodology for determining the TTs rely on the data of the agricultural census, conducted every 10 years and at the municipal household level. However, the biome undergoes important changes, observed by official monitoring systems: the Real-Time Deforestation Detection System (DETER, acronym in Portuguese) and Amazon Deforestation Monitoring Program (PRODES, acronym in Portuguese), monthly and yearly, respectively ^13^. Changes in land use and cover in deforested areas are observed by the TerraClass system (https://www.terraclass.gov.br/) ^14^. These data derived from images have spatial resolutions of 30m in PRODES, 10m in TerraClass 2020, and 30m in the other years of TerraClass. These elements are considered to estimate the minimum spatial unit of mapping − 2.5 hectares (ha) in these two systems − which can be aggregated in municipal totalizations, for example ^15^.

On the other hand, data from health information systems, especially from surveillance and monitoring systems such as the Brazilian Information System for Notificable Diseases (SINAN, acronym in Portuguese) and the Malaria − Epidemiological Surveillance Information System (Sivep-Malária, acronym in Portuguese), among others, were structured to support the design and evaluation of local, municipal, state, and national policies and programs in public health, as well as scientific studies ^11^ ^,^ ^16^ ^,^ ^17^. The minimum spatial unit of these systems is the “case”, centered on a specific address. To integrate health databases with environmental databases, the “case” can be aggregated to the municipal level, or to other levels, according to the analysis and specific periods. This integration is important because Amazonian municipalities present large territorial extensions and hold a diversity of socioenvironmental settings that have different possibilities for the analysis of the evolution of health-disease processes. To promote integrated health-environment-economy analyses in the Amazon, one must evaluate the state of TTs at least every year or every two years, in a more disaggregated spatial unit, appropriately located between the municipality and the “case”.

The technical systems associated with TTs transform the forest landscape and leave marks that can be captured on satellite images and be associated with other information from primary and secondary data ^7^. In this perspective, the representation of landscapes, associated with these technical systems and their TTs, constitute a synthesis territorial representation of the landscapes of production present in the regional agrarian economy. These mapped landscapes constitute a representation of the territories of production, which are territories of life and experiences of the agents involved in this economy.

The territories of production can be represented as a set of landscape units that capture the characteristics of technical systems associated with rural production and constitute part of the TTs. These landscape units can be identified and mapped with new approaches to classification of satellite-derived products, exploring machine learning with supervised classification and decision tree-based methods ^18^. We call these classified units as PLU. The PLU are the integrating territorial element, the structure this article proposes for integrated studies of health-disease processes considering the triad of health, environment, and economy in the Amazon.

To show the feasibility of the PLU identification and mapping methodology, we conducted a case study in the municipalities of Mocajuba and Cametá, located in the Baixo Tocantins region, in the State of Pará, Brasil, for 2021. We describe in this article the proposed methods to identify and map the PLU located in the municipalities, in a spatial unit of intra-municipal reference, and how to associate them with the types of TTs present in the agrarian economy of the Amazon. Thus, we seek to promote a debate on the use of PLU in the structuring of integrated approaches in health, associated with agrarian territories of production on an intra-municipal basis.

## Materials and methods

### TTs and the agrarian economy in the Amazon

The economist Francisco de Assis Costa introduced a new economic category for regional analysis: the TTs. According to the author ^9^ ^,^ ^10^, the technical solutions adopted at the level of production units are empirical materializations of TTs. These, in turn, are aggregates or convergences of these practices guided by a technological paradigm: “*a ‘model’ or ‘standard’ of solutions of technological problems, selected based on principles derived from the natural sciences and on selected material technologies*...” ^19^ (p. 152). Costa ^10^ highlights that the rural reality of the Amazon is characterized by a historical-geographical-cultural structural diversity. In this context, the evolutionary patterns related to the workforce, technologies, inputs, knowledge, and institutions that make up the agrarian economy of the Brazilian Amazon brings to light a set of TTs ^4^.

The TTs, guided by technological paradigms associated with production, combine agents and their microeconomic rationalities under two large groups: agroextractivist and mechanical-chemical-genetic ^20^. These paradigms guide different arrangements for the organization of their workforce, land structure, capital, and the package of technological solutions in their relations with the forest. Associated with these paradigms are two main economic agents: peasant and capitalist. While the agro-extractive paradigm is associated with the idea of a living nature, in which the forest is a means of production, the mechanical-chemical-genetic paradigm is associated with the idea of a dead nature, in which the forest is input, a resource to be consumed.

Costa ^9^ ^,^ ^10^ identified six TTs present in the regional agrarian economy, three of which are associated with peasant agents and three with capitalist. Box 1 shows a synthesis of these TTs and describes the patterns and arrangements that can be observed for the elements present in the landscape, shaped by these agents and their productive technical systems.

**Box 1:** Description of techno-productive trajectories (TTs) in association with elements present in the modified landscape.

TTS	AGENT	CHARACTERIZATION OF TTS	LANDSCAPE
TT1 Cultivation system (*temporary agriculture*)	Peasant	Based on an *agricultural paradigm*, this TT is associated with a diverse system of temporary crops such as cassava, pineapple, maize, and rice. We can characterize it as a diversified and relatively specialized peasant techno-productive trajectory, in which agricultural production is predominantly conducted by family units	Diverse landscape, including combinations of temporary small-scale agriculture, different stages of secondary vegetation, reduced areas of pasture, and continuous extensions of forests
TT2 Agroforestry		Based on an *agro-extractive paradigm*, this TT adopts a diverse approach in the use of natural resources, aiming to preserve the natural resources associated with the Amazon biome. The predominant activity is non-timber extractivist practices, which is based on agroforestry systems (AFSs), and it may or may not be interspersed with small areas destined for permanent agriculture, forestry, and temporary crops. Rural production is predominantly driven by family units	Landscape characterized by the predominance of primary forest, with patches of secondary vegetation, small-scale agriculture, and small areas of pasture
TT3 Cattle farms		Based on an *agribusiness paradigm*, this TT stands out for its extensive land use, with beef cattle as its core activity, possibly accompanied by dairy farming and temporary farming. This system is mainly based on family workforce, with a relatively low level of specialization compared to TT4	Landscape characterized by small and medium-sized pastures, some areas with shrubs. Reduced areas of small-scale agriculture, which may contain areas with vegetation in early stages, and forest fragments
TT4 Cattle farms	Capitalist	Based on an *agribusiness paradigm*, this activity has extensive use of land for beef cattle breeding, presenting a higher degree of specialization compared to TT3. The execution of this activity involves salaried labor, but with a relatively low employment rate	Homogeneous landscape, characterized by extensive and uniform areas of managed pastures, with isolated fragments of primary and secondary forest
TT5&6 Permanent agriculture (silviculture)		Based on an *agribusiness paradigm*, TT5 refers to enterprise monoculture specializing in permanent agriculture and adopts a land-use intensive approach. On the other hand, TT6 has its basis in silviculture. Both TTs depend on salaried labor and the application of *mechanical-chemical-genetic* methods	Homogeneous landscape formed by the cultivation of a limited number of tree and shrub species, and isolated fragments of primary forest may occur. In the case of managed forest, selective logging can occur
TT7 Annual agriculture		Based on an *agribusiness paradigm*, this TT is based on temporary agriculture, specializing mainly in the production of grains such as soybeans and maize. It makes intensive use of the soil, incorporating mechanic-chemical-genetic technologies and requiring the use of salaried labor	Homogeneous landscape, characterized by extensive and homogeneous areas of agricultural cultivation, and isolated fragments of primary forest may or may not occur

Source: adapted from Costa ^4^ ^,^ ^9^ and Codeço et al. ^11^.

### Identifying the production landscape units

The first methodological step consists in choosing the resolution of the cell. Each cell represents a landscape unit, and their set forms a grid (cell space) ^7^. Land use and cover information retrieved from satellite imagery is generally provided systematically, with 10m, 20m, and 30m of average spatial resolution. Thus, to evaluate the spatial arrangement and composition of land use and cover classes that make up production landscapes, we worked with a cell grid, with a [2×2]km spatial resolution. The definition of cell size is done empirically ^7^ and involves the use of medium-resolution satellite imagery, land cover use data, and field records, integrated and analyzed in a Geographic Information System (GIS). The definition of the cells size depends on the characteristics of the region, objects, and categories of interest ^7^. Therefore, we considered the size of the areas of agricultural use that vary in the study area, from 0.5 to 2ha for peasants and above this value for medium and large agricultural capitalist systems, reaching 200ha or more. Based on the defined cell size, 1,291 cells were generated for the study area: 290 for the municipality of Mocajuba and 1,001 for Cametá.

The construction of the PLU is influenced by the cells size that will delimit the patterns of the landscapes of interest. The PLU is described based on the observation, in the databases, of the spatial arrangements and the composition of the landscape elements (polygons and classes of land use and cover) found in the cell, in addition to the context information, such as density and size of rural establishments, proximity to rivers, location on island or floodplain areas. For this construction, we start from the principle that the technical systems of rural production present landscapes with specific spatial patterns, visually identifiable in the data derived from satellite images, showing correspondences with the TTs, but not necessarily in a direct relationship.

To build the typology of the PLU for the region, it is necessary to consider its economic and agrarian context. The municipalities of Cametá and Mocajuba are located in the Baixo Tocantins region in Pará State, the second largest *açaí* producing region in Pará ^21^. This region has significant agro-extractive and agricultural production, based on peasant technical systems. *Açaí* is produced in different systems, in areas of islands, floodplains, and, more recently, in areas of upland ^22^. Small-scale agriculture, also presenting with an expressive economy, is located in areas of upland, associated with the cultivation of black pepper, cocoa, and cassava, the latter produced in a fallow system ^22^ ^,^ ^23^. Besides the peasants and their technical systems, there are capitalists and their modes of production, especially associated with the production of *açaí* in upland. In addition to the *açaí* produced in irrigated and mechanized systems, there is the production of palm oil, black pepper, and cocoa, the latter two often consorted with *açaí* ^22^. Fish farming is also observed, developed along with agricultural production, in both capitalist and peasant systems. Notably, this area, unlike most regions of the Amazon, has little livestock activity ^22^ ^,^ ^24^.

To build a typology representing the PLU categories present in the region for 2021, an important information was incorporated into the land use and cover data: the indicator of potential areas of occurrence of native *açaí* palms. Given the diverse nature of the *açaí* productive systems, the available systematic production data of land use and cover, such as those of TerraClass ^14^ and MapBiomas (https://brasil.mapbiomas.org/) ^25^, were not used, since they do not identify the small-scale agriculture nor do they differentiate stages of secondary vegetation, largely associated with fallow shift cultivation. The need for better identification of crops led to the classification of land using Sentinel-2A (https://www.engesat.com.br/sentinel-2/) satellite images with 10m spatial resolution, to obtain classes of use and coverage more detailed and adhering to the characteristics of the study area.

Image data cubes from Sentinel-2A ^26^ were used for classification, as well as supervised classification using Random Forest ^27^, implemented in the SITS package (Satellite Image Time Series Analysis on Earth Observation Data Cubes) ^26^. The Cube was produced with monthly images from January, 1st 2021 to Dicember, 31 2021. Ten spectral bands were used, except the water vapor band. A map of land use and cover was generated, presenting 94% overall accuracy and a 0.92 kappa coefficient and containing the following classes: (a) small-scale agriculture; (b) large-scale agriculture; (c) herbaceous pasture; (d) shrubs pasture; (e) initial secondary vegetation; (f) advanced secondary vegetation; and (g) hydrography. The final map includes the forest and non-forest classes from PRODES ^13^.

Indicator of potential areas of occurrence of native *açaí* palms is prepared from the combination of forest cover data with data from hydro-topographic environments ^28^. A hydro-topographic map is generated to indicate the lowland areas, transition zones (between floodplains and slopes), slope areas, and plateau areas (upland). The data generated by hydro-topographic mapping were combined with forest cover data. Thus, the areas with high potential for occurrence of native *açaí* (*Euterpe oleracea*) were defined as having primary or secondary forest cover, in floodplain (lowland) areas or in transition zones. Moderate potential areas were identified in zones with forest cover in slope areas; low potential areas were identified by presenting secondary vegetation and/or forest on upland ^24^. The areas that did not present primary forest or secondary vegetation were defined as areas of absence of *açaí* palms. The class of high potential of occurrence of *açaí* palms was integrated with land use and cover data, forming a new unified database. This integration is important in determining the types of PLU present in the constructed typology.

**Figure 1:**
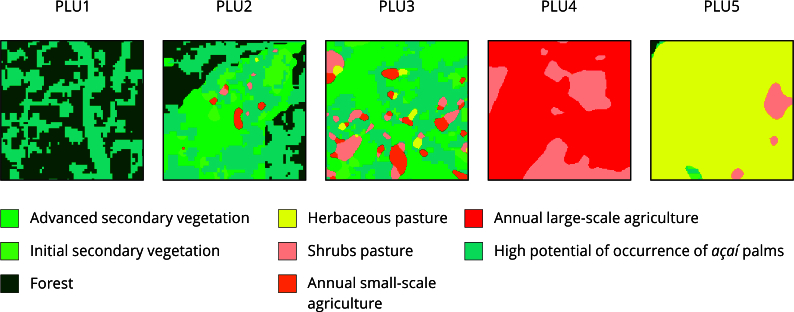
Patterns of production landscape units (PLU) observed with land use and land cover data using [2×2]km cells.

PLU1: extractivist peasant unit; PLU2: agro-extractivist peasant unit with temporary agriculture; PLU3: peasant unit with *açaí* production, permanent and/or temporary agriculture, and pastures; PLU4: capitalist unit with irrigated planted *açaí*; PLU5: capitalist unit with cattle farms.

The unified land use and cover database was added to the [2×2]km cells, making it possible to define five types of PLU found in the study area. Three PLU were associated with peasant systems and two with agricultural capitalist systems, namely: (1) extractive peasant unit - PLU1; (2) agro-extractive peasant unit with temporary agriculture - PLU2; (3) peasant unit with *açaí* production, permanent and/or temporary agriculture, and pastures - PLU3; (4) capitalist unit with irrigated planted *açaí* - PLU4; and (5) capitalist unit with cattle farms - PLU5. Figure 1 illustrates the spatial patterns relative to the new land use and cover map, which characterize the types of PLU identified in the study area.

The PLU characterized in this study were developed considering that the technical systems associated with the production of *açaí* are predominant in the region. However, in upland environments, one can find other types of crops such as black pepper and palm oil, which may or may not include *açaí*, in addition to livestock areas that do not include *açaí*. As the PLU are inserted in different landscapes, such as island, floodplain, and upland environments, the crops and technological practices adopted in the management, harvesting, and storage of production are different. Therefore, the relations of these PLU with risk/protective factors in the context of local health are also different. Box 2 shows the final typology, covering the five types of PLU.

**Box 2:** Typology of the production landscape units (PLU) associated with the different technical systems of agricultural production and their correspondence with the analytical economic categories described by the techno-productive trajectories (TTs).

TYPE	LANDSCAPE STRUCTURE, COMPOSITION, AND CONTEXT	CHARACTERIZATION OF THE PLU	TT
PLU1 Extractivist peasant unit	Areas with a predominance of classes with *high potential of occurrence of açaí palms* and spots of *forest*, may contain isolated patches of *advanced secondary vegetation* **Context**: region of islands or near the Tocantins River. Cells with a high density of rural establishments, the plots do not exceed 280ha	System based on an *agro-extractive paradigm.* This PLU is characterized by an *Agroforestry System* (AFSs-F) originating in the biome. In this unit, *açaí* extractivist practices, which stands out as the main economic activity, can be complemented by fishing. As a secondary product, one can find cocoa planted near the *açaí* areas. Family labor predominates (more than 50%), supplemented by daily workers during the *açaí* collection period	TT2
PLU2 Agro-extractivist peasant unit with temporary agriculture	Diversified landscape. Areas with a predominance of classes with *high potential of occurrence of açaí palms* and *forest,* including small plots of *small-scale agriculture, advanced secondary vegetation,* and small fragments of *initial secondary vegetation* **Context:** floodplain region of the Tocantins River and banks of streams. Cells with a high density of rural establishments, the plots do not exceed 280ha	Based on an *agro-extrativist paradigm*, this PLU is characterized by an *Agroforestry System* (AFSs-A) combined with forest management, especially *açaí* with agricultural practices. In this unit, the *açaí* economy is complemented by temporary agriculture, carried out in areas not affected by seasonal flooding of rivers, such as cassava with the use of fallow systems. As a secondary product, one can find cocoa planted near *açaí* areas or on upland, as well as black pepper. The labor force is predominantly family and can be supplemented by day workers during the *açaí* collection period	TT2
PLU3 Peasant unit with *açaí* production, permanent and/or temporary agriculture, and pastures	Diversified landscape. Areas characterized by a mosaic of classes with *high potential of occurrence of açaí palms*, *small-scale agriculture*, spots of *initial secondary vegetation*, which may include small isolated areas of clean and shrubs pasture with an irregular shape **Context:** upland region. They are more distant from the floodplains and islands of the Tocantins River. Cells with a moderate density of rural establishments, the plots do not exceed 280ha.	Based on an *agribusiness paradigm*, this PLU stands out for presenting a diverse system of temporary and permanent cultures, with some specialization. *Açaí*, along with the production of black pepper and cassava, constitutes one of the relevant activities, without necessarily being the main activity in terms of area and production value. The production of *açaí* can occur in consortium with a set of permanent crops, such as black pepper and cocoa. Cassava cultivation in fallow systems and flour production are important activities for both consumption and household income. It may present fish farming tanks. Production is mostly conducted by family labor	TT2
PLU4 Capitalist unit with irrigated planted *açaí*	Homogeneous landscape. Areas with predominance of class *large-scale agriculture* with regular geometric shape. They may present isolated spots of *secondary vegetation at different stages* and pasture areas with well-defined geometric shapes **Context:** upland region, distant from the Tocantins River. Cells with low density of rural establishments, and the plots are larger than 280ha	Based on an *agribusiness paradigm*, this PLU stands out for the more intensive use of land, adopting a system specialized in enterprise agriculture based on the *mechanic-chemical-genetic* model. Although it has its bases in the production of *açaí*, it can also integrate other crops such as black pepper, cocoa, and *cupuaçu*. Black pepper and cocoa can be grown in consortium with *açaí*. Palm oil extraction and fish farming may or may not occur. Production is mostly conducted by salaried labor	TT5&6
PLU5 Capitalist unit with cattle farms	Homogeneous landscape. Areas with predominance of classes *herbaceous pasture* and *shrubs pasture* with well-defined geometric shape. Isolated fragments of *forest* or *advanced secondary vegetation* may occur **Context:** upland region, distant from the Tocantins River. Cells with low density of rural establishments, and the plots are larger than 280ha	Based on an agricultural paradigm, this PLU is characterized by extensive land use in a livestock-oriented business system, predominantly driven by salaried labor	TT4

### Mapping the production landscape units

The classification of the PLU was based on the typology developed (Box 2). Spatial attributes, composition, and context are extracted for each cell of [2×2]km for the classification of PLU, in which we use a supervised classifier based on decision trees with boosting method ^18^. The attributes, or landscape descriptors, are used to train the algorithms that will perform PLU classification in a supervised manner. The sample collection process is carried out iteratively, until a consistent set of training samples is obtained to adequately represent the elaborated typology. The boosting method is used to improve classification accuracy by creating multiple decision trees based on a predefined number of iterations, in which at each iteration, the adjustment of training errors that occurred in the previous iteration is performed ^18^. Combining several trees, with different metrics of composition, structure, and context of the landscape, the final decision of the model results from a voting system, which assigns to each cell the most frequent class imputed by the decision trees ^7^ ^,^ ^18^.

The classification of the PLU consisted of four steps and was carried out within the integrated environment Geographic Data Mining Analyst-GeoDMA ^29^:

i) Attribute extraction. Using land use and cover data, 246 metrics of landscape composition, structure, and context were extracted, such as distance to rivers, distance to roads, presence/absence of rivers, number and size of rural establishments, for each cell;

ii) Selection of training and test samples. A total of 212 samples were collected: 48 samples for PLU1, PLU2, PLU3, and PLU5 and 20 samples for PLU4. The lower number of samples for PLU4 is due to the lower representativeness of cells with the corresponding spatial pattern. Among the 212 samples collected, 70% were intended for classifier training and 30% were used in the validation phase as test samples;

iii) Classification of cells by boosted decision tree algorithm, with 99 iterations and independent classifications;

iv) Classification assessment. The assessment was performed using a confusion matrix with the set of test samples.

## Results

**Figure 2:**
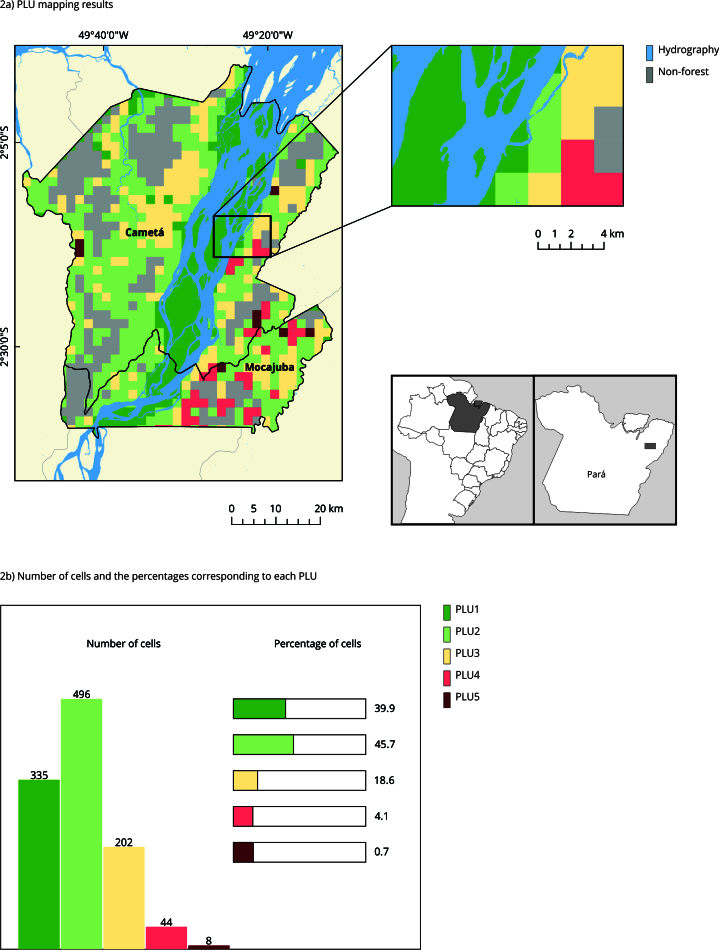
Mapping results and enlarged area with details of the production landscape units (PLU), and the number of cells and percentages corresponding to each PLU present in the study area. Brazilian Amazon, 2021.

PLU1: extractivist peasant unit; PLU2: agro-extractivist peasant unit with temporary agriculture; PLU3: peasant unit with *açaí* production, permanent and/or temporary agriculture, and pastures; PLU4: capitalist unit with irrigated planted *açaí*; PLU5: capitalist unit with cattle farms.

Figure 2a presents the PLU mapping result, including an enlarged area. The peasant PLU (PLU1, PLU2, PLU3) cover 95% of the cells, evincing the dominance of the economic activities of these agents in the studied region (Figure 2b). PLU2 represents more than 45% (496 cells) of the total mapped cells, followed by PLU1, corresponding to almost 31% (335 cells), and PLU3 with 18% (202 cells). PLU4 corresponds to 4.1% (44 cells) while PLU5 does not reach 1% (8 cells). In alignment with the TTs, PLU1 and PLU2 are associated with the TT2, representing together almost 77% of the total mapped in the region, corroborating Nogueira et al. ^23^, and field records ^22^, that indicate the predominance of activities of peasants in the region. Considering the last Agricultural Census (2017), the peasant trajectory TT2 is dominant in both municipalities. Regarding the capitalist trajectory, TT5&6 prevails in Cametá and TT7 prevails in Mocajuba ^12^.

**Figure 3:**
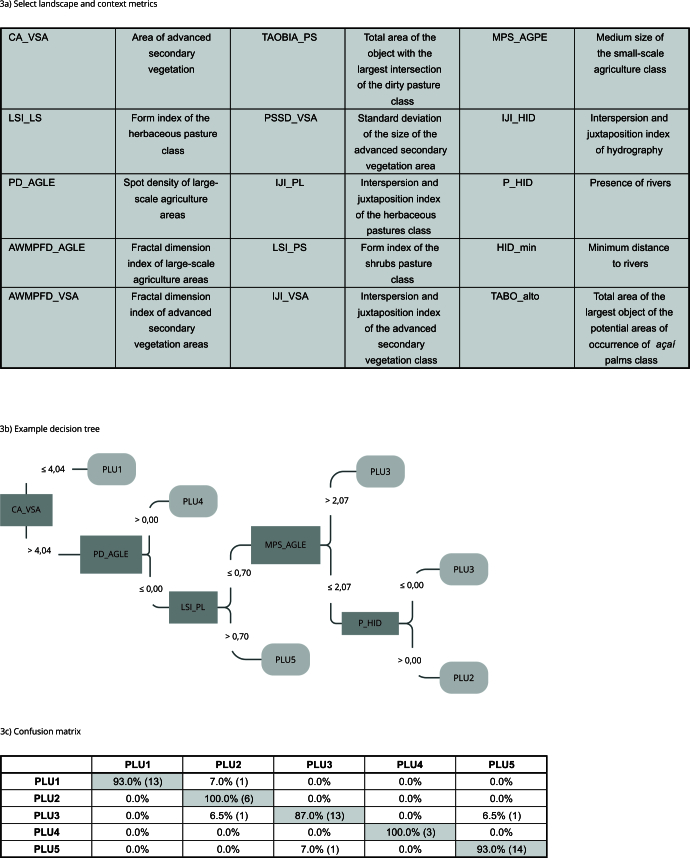
Description of the 15 metrics used in the classification of production landscape units (PLU), decision tree and confusion matrix for PLUs classified using test samples.

PLU1: extractivist peasant unit; PLU2: agro-extractivist peasant unit with temporary agriculture; PLU3: peasant unit with *açaí* production, permanent and/or temporary agriculture, and pastures; PLU4: capitalist unit with irrigated planted *açaí*; PLU5: capitalist unit with cattle farms.

In supervised classification ^18^, six decision trees were generated to classify the PLU. The algorithm parameterization predicted 99 iterations, however, it was stabilized in the sixth training cycle. The adjustment error of the model varied from 0.6% to 4.4% until it stabilized in the last iteration at 0%. The choice of attributes is performed by an entropy measure that estimates the information gain of the variables during classification, discarding those with lower gains. Figure 3a shows the selected attributes. Of the 15 metrics used in the decision trees, four were used at the root, generating the initial branches: (1) area of advanced secondary vegetation (CA_VSA); (2) form index of the herbaceous pasture class (LSI_PL); (3) spot density of large-scale agriculture areas (PD_AGLE) and; (4) fractal dimension index of large-scale agriculture areas (AWMPFD_AGLE). Most metrics were used to differentiate PLU4 or PLU5 from the others, with the exception of CA_VSA, which separated PLU1 in the first cut. In the intermediate and final branches of the trees, other metrics stood out, such as medium size of small-scale agriculture class (MPS_AGPE), in addition to three context metrics related to minimum distance to rivers (HID_min), presence of rivers (P_HID), and hydrography interspersion and juxtaposition index (IJI_HID), used to separate PLU2 from PLU3. PLU2 is located near the rivers, in which activities such as *açaí* management and collection predominate, with smaller areas dedicated to agriculture, while PLU3, located farther from the rivers, has agriculture as the main economic activity, and may or may not include *açaí* crops.

In one of the trees, the TABO_alto metric was used, which measures the Area of the largest object in the class high potential for the occurrence of *açaí* palms. This metric separated PLU3 from PLU2. This result shows the importance of differentiating the classes of agriculture in the input data for the distinction of capitalist and peasant PLU. No metrics of landscape composition, density, and size of agricultural establishments were used. Improvements and updates to the input databases could improve, for example, the separation between peasant units and capitalist units. Figure 3b shows one of the six trees generated with the landscape structure and context metrics.

The confusion matrix of Figure 3c enables an evaluation of the classification with 30% of the test samples. There were few confusions, and the most significant occurred between PLU3, peasant, and PLU5, capitalist associated with livestock. This occurred because both PLU3 and PLU5, located on upland, can present classes such as shrubs pasture, initial secondary vegetation, and forest. Imbalance in training samples is a limitation of this method. PLU1 was confused with PLU2 in 7% of the cells. There were no confusions between PLU2 and PLU4, although PLU4 presents a limited number of samples.

## Discussion

The classification is consistent with the expectations for this region, aligning with field records ^22^ and works incorporating the TTs ^11^ ^,^ ^23^. The mapping of PLU at the intramunicipal scale showed the persistence in 2021 of peasant TTs associated with PLU1, PLU2, and PLU3. PLU1 and PLU2 predominate in the islands and floodplains of Baixo Tocantins and its tributaries. The presence of PLU3 is identified as it moves away from floodplain areas. PLU4 and PLU5, associated with capitalist TTs, although less expressive, are more distant from Baixo Tocantins, located close to roads and highways, in upland areas, in which activities such as livestock and *açaí* monoculture are developed, configuring homogeneous landscape patterns. The PLU associated with the TTs coexist and interact in different ways in the economic dynamics of rural Amazon. The changes in the structure and composition of forest landscapes of the ways of living and modes of production, associated with technical systems, are captured by the PLU. Thus, the PLU constitute a structure of representation that introduces new possibilities to (re)think the elements of exposure and contact of populations in these areas to old and new pathogens ^30^, along with the integrated health-environment-economy approaches.

Diseases such as visceral leishmaniasis (VL), cutaneous leishmaniasis (CL), malaria, dengue, Chagas disease, for example, prevail in the Amazon to the present day ^11^ ^,^ ^31^. The *Trajetorias* ^12^ database, constructed for all the municipalities of the Brazilian Legal Amazon, presents the incidence rates of the main diseases found in the studied area, stratified by urban and rural areas. In Cametá, the *Trajetorias* ^12^ database shows, from 2015 to 2019, an incidence of malaria (*Plasmodium vivax*) of 4,620.19 in the rural environment, considering the rate per 100,000 inhabitants. Chagas disease had a rate of 22.61, while VL had 27.60 and CL, 6.65. In Mocajuba, no cases of Chagas disease were registered from 2015 to 2019 in the rural environment. The incidence rate of malaria (*Plasmodium vivax*) was 2,466.58, VL was 31.54, and the CL, 40.15.

PLU1 and PLU2 present socio-environmental conditions that favor the occurrence of malaria (*Plasmodium vivax*), Chagas disease, and CL in the study area. In communities with *açaí* production in Baixo Tocantins, cases of Chagas disease with oral transmission were recorded ^32^. Generally, it is in island and floodplain areas that peasants grow *açaí*, represented by PLU1 and PLU2. The processes of fruit harvesting, sanitation, and storage introduce risks for oral transmission, since the fruits can be contaminated with feces and urine of the vector, containing *Trypanosoma cruzi* ^33^, at any of these stages. Although farmers receive guidance by agents of local institutions and by the industries that buy the *açaí* fruit, in practice they are still insufficient for effective control of the disease, resulting in the incidence of Chagas in the region. In the case of CL, the study by Sousa Júnior ^31^ shows that from 2007 to 2016, islands and floodplains areas in Cametá had the highest numbers of CL cases.

Leishmaniasis is predominantly transmitted by the bite of sandflies (*birigüi*, mosquito-palha) of the genus *Lutzomyia*. These mosquitoes use moist places, with shade and organic matter, such as leaves and fruits, as well as animal feces, to reproduce. Animal burrows, the base or trunk of trees are potential breeding grounds. They feed on the blood of various species of wild animals, synanthropic animals, and, in specific transmission cycles, domestic animals ^31^. These can be natural hosts or (potential) wild reservoirs. PLU1 and PLU2 present situations of houses very close to the forest and forest environments close to the home environment and production areas, and there is also the presence of domestic and wild animals, in particular rodents. These are landscapes that increase the risks of exposure and contact of sandflies with peasant producers. Guidelines related to the management of production territories, which in the case of peasants involves their home and peridomicile, such as cleaning backyards and their cultivation and extraction areas, can be a strategy designed by health surveillance, integrated with other municipal policies.

PLU3, although peasant, presents a differentiated technical system. It is located in more diverse landscapes, with *açaí* production, permanent and/or temporary agriculture, and pastures. It presents socio-environmental conditions that favor the risk of CL and VL transmission. It occurs on the mainland, encompassing the urban fringes, distant from rivers but close to vicinal roads. It is located in regions where forest ecosystem disturbances are more pronounced than in PLU1 and PLU2. Deforestation, proximity to vicinal roads, and proximity to dense urban areas, characteristics of these PLU, increase the risks for leishmaniasis (cutaneous and visceral), as observed in Baixo Tocantins ^31^ ^,^ ^34^. In the case of this PLU, the issues related to the processes of the *açaí* production chain, collection, sanitation, and storage, associated with Chagas disease, are also present ^33^.

PLU4 is associated with *açaí* monoculture. This PLU has a technological solution package with the use of mechanics, chemistry, genetics, with specialized technical assistance. However, it produces a little diverse environment, with little forest cover. The circulation of wildlife is restricted to nearby forest fragments and the new “forest” of planted palms, in general, intercropped with another culture. This environment favors the transmission of diseases that have wild animals in their cycle, which are hosts and reservoirs of a wide range of parasitic species. *Açaí* in consortium with cocoa presents socio-environmental conditions favorable to contamination by VL ^34^. On the other hand, the population density in these areas is lower, since it employs little labor, reducing the chances of contact and risks of transmission on site or in neighboring areas.

PLU5 is characterized by extensive livestock farming. The technical systems of extensive livestock farming significantly consume the forest cover, which is replaced by pasture. Thus, they draw transition zones between pasture areas and forest areas, which become conducive habitats for wild rodents ^35^. The landscapes of livestock production in Cametá and near the banks of the Tocantins River recorded occurrences of CL ^31^. For these territories, of types PLU4 and PLU5, health surveillance can design specific actions and establish, together with other municipal public sectors and with capitalist, procedures to monitor and control the presence and circulation of wild animals, in the interest of local public health.

In this analysis, still initial and descriptive, we observed how to identify, characterize, and map the PLU, helping to understand the differentiated risks of human exposure to pathogens. An analytical framework that integrates socio-environmental elements in the context of agricultural production is a promising structure to be investigated. Although these PLU have been mapped for only one year, in a specific region, they can be produced for several years and adapted to other regions, allowing one to observe the dynamics of technical systems and TTs ^7^, their evolution, and in which directions their dynamics change the landscapes of the biome, at the intra-municipal scale. Spatial data, derived from satellite images, make it possible to generate refined analyzes at different spatial and temporal scales ^7^ ^,^ ^24^.

In this article, we did not explore PLU as a variable in statistical or mixed models to study the relationships between its dynamics and the emergence, re-emergence, or intensification of health-disease processes. Our focus was to point out this new integrative structure as a territorial representation of the health-environment-economy triad and its potentials in integrated health studies. Understanding the interaction between the social agents that promote economies in the biome, the landscapes they transform, and their impacts on health-disease processes is essential to develop (new) surveillance and control strategies or rethink existing ones. In this process, one should consider the constant transformation of the forest ecosystem, driven by the decisions of its economic agents, and their impacts on public health ^11^ ^,^ ^16^, affecting the communities and all Amazonian populations.

The structure presented can help to instrumentalize the debates in the health dimension regarding policies and proposals for more effective strategies, associated with choices of paths for agrarian economic development in the Brazilian Amazon. At the moment, a dispute over Bioeconomy ^20^ projects is underway as a strategy for regional development. Understanding what Bioeconomy we are talking about and, in particular, how our choices for one project or another impact the health of the forest ecosystem and the health of populations in the Amazon, is necessary and urgent.
