# Supplementary material for: Integrated health-environment-economy approach in the Brazilian Amazon: mapping production landscape units
Source: Cad Saude Publica. 2025 Mar 24;41(Suppl 1):e00066424. doi: 10.1590/0102-311XEN066424 (PMC11940853; doi:10.1590/0102-311XEN066424)
Supplement: Translation

# Translation: Abordagem integrada em saúde-ambiente-economia na Amazônia brasileira: mapeando unidades de paisagem da produção

**DOI:** 10.1590/0102-311XPT066424

**Authors:** Anielli Rosane de Souza, Maria Isabel Sobral Escada, Ana Paula Dal’Asta, Marcus Vinicius Gonçalves da Silva, Danilo Araújo Fernandes, Antonio Miguel Vieira Monteiro

**Affiliations:** 1 Instituto Nacional de Pesquisas Espaciais, São José dos Campos, Brasil.; 2 Universidade Federal de Lavras, Lavras, Brasil.; 3 Universidade Federal do Pará, Belém, Brasil.

**Keywords:** Tecnologia de Sensoriamento Remoto, Ecossistema Amazônico, Economia Rural, Vigilância em Saúde Pública, Classificação

## Abstract

A economia agrária, com seus agentes sociais e sistemas técnicos, mobiliza os elementos que geram as transformações nas paisagens social e florestal na Amazônia brasileira. As escolhas para o desenvolvimento regional levam à sustentabilidade ou insustentabilidade do ecossistema florestal e de sua paisagem social. A saúde é negligenciada nesse debate. Argumentamos que uma estrutura analítica para abordagens integradas, saúde-ambiente-economia, necessita de uma representação territorial para as paisagens associadas aos modos de viver e produzir no agrário amazônico, as unidades de paisagem de produção (PLU, acrônimo em inglês). Neste artigo, exploramos técnicas de aprendizado de máquina, no campo da classificação supervisionada, com métodos baseados em árvores de decisão, para identificar e mapear as PLU. Um estudo de caso foi desenvolvido para os municípios de Mocajuba e Cametá, na região do Baixo Tocantins, no Estado do Pará, para o ano de 2021. Descrevemos como identificar e mapear as PLU em uma unidade espacial de referência intramunicipal e como associá-las aos tipos de trajetórias tecnoprodutivas rurais ou trajetórias tecnológicas (TTs) presentes na economia agrária regional, bem como promovemos uma discussão inicial do uso das PLU na estruturação de abordagens integradas em saúde. Este artigo contribui para alinhar debates sobre estratégias para o desenvolvimento econômico à promoção da saúde na Amazônia brasileira.

## Introdução

Abordagens analíticas para processos saúde-doença na Amazônia brasileira devem oferecer uma estrutura que integre a tríade saúde, ambiente e economia. A economia agrária ^1^, com seus diversos agentes e sistemas técnicos, é o principal fator que mobiliza os elementos que geram as transformações nas paisagens social e florestal do bioma. Essas transformações produzem distúrbios de origem antrópica no ecossistema florestal e afetam sua integridade estrutural e funcional ^2^, alterando os padrões de uso e cobertura da terra, o papel das unidades socioespaciais e seu arranjo no urbano contemporâneo Amazônico ^3^. Alteram também a distribuição da renda, dos bens e serviços associados às cadeias envolvidas nas economias regionais e locais. Nesta perspectiva, esses sistemas técnicos de produção estão diretamente relacionados à escolha de um caminho de desenvolvimento regional que pode levar à sustentabilidade ou insustentabilidade da paisagem florestal e da paisagem social, interligadas na história de ocupação da Amazônia brasileira ^4^ ^,^ ^5^. Nesse contexto, a dimensão da saúde é pouco incluída nessas discussões. Neste artigo, argumentamos que, para promover essa estrutura analítica, é necessário uma nova representação territorial para as paisagens do bioma, organizadas como unidades de paisagem de produção (PLU, acrônimo em inglês). Estas são unidades associadas aos modos de produzir operados pelos sistemas tecno-produtivos rurais, presentes na economia agrária.

A PLU é concebida como uma representação para o estado das paisagens florestais modificadas pelos sistemas técnicos associados à produção rural. No contexto amazônico, as PLU abrangem uma variedade de ecossistemas e ambientes que se intercalam e se sobrepõem em decorrência das interações entre os agentes de produção, como agricultores, extrativistas, pecuaristas e comunidades locais, e os componentes da paisagem, como rios, florestas, estradas e cidades. Esses elementos são organizados em arranjos espaciais variados e não uniformes, refletindo a diversidade e a complexidade das interações sociais, econômicas e ambientais ^6^ ^,^ ^7^ ^,^ ^8^. Uma vez identificadas e mapeadas, as PLU podem ser usadas como *proxy*, ou seja, como uma variável indicadora substituta, para os tipos de sistemas técnicos associados à produção rural, no recorte territorial em análise. As trajetórias tecnoprodutivas rurais ou trajetórias tecnológicas (TTs) são categorias analíticas introduzidas por Costa ^9^ ^,^ ^10^ para a análise da economia agrária e representam padrões de soluções técnicas e produtivas que se constituem como trajetórias específicas, adotadas por diversas unidades de produção rural, camponesa ou patronal.

Os padrões observados nas paisagens modificadas são influenciados pelos modos de viver e produzir, associados às escolhas dos modelos de produção dos agentes camponês e patronal. A análise de padrões espaciais específicos, observados em paisagens modificadas, possibilita associá-los às PLU e às TTs correspondentes. Como as PLU representam paisagens constantemente moldadas pelos sistemas técnicos de produção, esses padrões espaciais, tipicamente presentes nos espaços de vida e vivência desses agentes, podem ser identificados e mensurados em imagens de satélite.

Codeço et al. ^11^ destacam a importância de abordagens integradas, considerando economia, ambiente e processos saúde-doença para (re)formular estratégias de vigilância em saúde. O artigo ^11^ oferece uma estrutura para debater as dimensões econômicas, ambientais e de saúde, em nível municipal.

A dimensão econômica é analisada a partir da categoria analítica definida por Costa ^4^ ^,^ ^9^ como descritora da economia agrária na Amazônia, as TTs. Costa ^10^, utilizando dados dos censos agropecuários (1995, 2006 e 2017), em uma metodologia baseada em regressões estatísticas, principais componentes e análise fatorial, revela a presença de seis tipos de TTs nos municípios da Amazônia brasileira. O estudo de Codeço et al. ^11^ associa a TT dominante na economia agrária municipal a indicadores ambientais e epidemiológicos a partir de uma base de dados organizada e harmonizada em nível municipal ^12^.

A metodologia para determinação das TTs depende dos dados do censo agropecuário, realizado decenalmente e em nível do agregado municipal. No entanto, o bioma sofre alterações importantes, observadas pelos sistemas oficiais de monitoramento: Sistema de Detecção de Desmatamentos em Tempo Real (DETER) e Projeto de Monitoramento do Desmatamento na Amazônia Legal por Satélite (PRODES), mensal e anual, respectivamente ^13^. As mudanças dos usos e coberturas da terra nas áreas desmatadas são observadas pelo sistema TerraClass (https://www.terraclass.gov.br/) ^14^. Esses dados derivados de imagens têm resoluções espaciais de 30m no PRODES, 10m no TerraClass 2020 e 30m nos demais anos do TerraClass. Tais elementos são considerados para estimar a unidade espacial mínima de mapeamento, de 2,5 hectares (ha) nesses dois sistemas, que pode ser agregada em totalizações municipais, por exemplo ^15^.

Por outro lado, os dados dos sistemas de informação em saúde, em particular os de vigilância e monitoramento, como os Sistema de Informação de Agravos de Notificação (SINAN) e Sistema de Informação de Vigilância Epidemiológica − Malária (Sivep-Malária), entre outros, foram estruturados para apoiar o desenho e avaliação local, municipal, estadual e nacional, de políticas e programas na saúde pública, assim como estudos científicos ^11^ ^,^ ^16^ ^,^ ^17^. A unidade espacial mínima desses sistemas é o “caso”, centrado em um endereço específico. Para integrar as bases de dados de saúde com as bases ambientais, o “caso” pode ser agregado para o nível municipal, ou para outros níveis, de acordo com a análise e períodos específicos. Essa integração é importante, porque os municípios amazônicos apresentam grandes extensões territoriais e contêm uma diversidade de situações socioambientais que abrigam diferentes possibilidades para a análise da evolução dos processos saúde-doença. A fim de promover análises integradas em saúde-ambiente-economia na Amazônia, é necessário avaliar o estado das TTs pelo menos a cada ano, ou a cada dois anos, em uma unidade espacial mais desagregada, adequadamente situada entre o município e o “caso”.

Os sistemas técnicos associados às TTs transformam a paisagem florestal e deixam marcas que podem ser capturadas em imagens de satélites e associadas com outras informações provenientes de dados primários e secundários ^7^. Nessa perspectiva, a representação das paisagens, associadas a esses sistemas técnicos e suas TTs, constituem uma representação territorial síntese das paisagens da produção presentes na economia agrária regional. Essas paisagens mapeadas constituem uma representação dos territórios da produção, que são territórios de vida e vivências dos agentes envolvidos nessa economia.

Os territórios da produção podem ser representados como um conjunto de unidades de paisagem, as quais capturam as características dos sistemas técnicos associados à produção rural e são parte constitutiva das TTs. Essas unidades de paisagem podem ser identificadas e mapeadas com novas abordagens para classificação dos produtos derivados de imagens de satélite, explorando técnicas de aprendizado de máquina com classificação supervisionada e métodos baseados em árvores de decisão ^18^. Denominamos essas unidades classificadas de PLU. As PLU são o elemento territorial integrador, a estrutura que este artigo propõe para estudos integrados dos processos saúde-doença, considerando a tríade: saúde, ambiente e economia, na Amazônia.

Para demonstrar a viabilidade da metodologia de identificação e mapeamento das PLU, realizamos um estudo de caso nos municípios de Mocajuba e Cametá, situados na região do Baixo Tocantins, no Estado do Pará, para o ano de 2021. Descrevemos neste artigo os métodos propostos para identificar e mapear as PLU presentes no município, em uma unidade espacial de referência intramunicipal, e como associá-las aos tipos de TTs presentes na economia agrária da Amazônia. Assim, buscamos promover uma discussão sobre o uso das PLU na estruturação de abordagens integradas em saúde, associadas aos territórios da produção agrária em base intramunicipal.

## Materiais e métodos

### As TTs e a economia agrária na Amazônia

O economista Francisco de Assis Costa introduziu uma nova categoria econômica para análise regional: as TTs. Para Costa ^9^ ^,^ ^10^, as soluções técnicas adotadas ao nível das unidades de produção são materializações empíricas de TTs. Estas, por sua vez, são agregados ou convergências dessas práticas orientadas por um paradigma tecnológico: “*um ‘modelo’ ou ‘padrão’ de soluções de problemas tecnológicos, selecionados com base em princípios derivados das ciências naturais e em tecnologias materiais selecionadas...*” ^19^ (p. 152). Costa ^10^ sublinha que a realidade rural da Amazônia é caracterizada por uma diversidade estrutural histórico-geográfica-cultural. Nesse contexto, os padrões evolutivos relacionados à força de trabalho, tecnologias, insumos, conhecimentos e instituições que compõem a economia agrária da Amazônia brasileira fazem emergir um conjunto de TTs ^4^.

As TTs, orientadas por paradigmas tecnológicos associados à produção, combinam agentes e suas racionalidades microeconômicas sob dois grandes grupos denominados agroextrativista e mecânico-químico-genético ^20^. Esses paradigmas orientam diferentes arranjos para a organização da sua força de trabalho, estrutura fundiária, do capital e do pacote de soluções tecnológicas em suas relações com a floresta. Associados a esses paradigmas estão dois principais agentes econômicos: camponês e patronal. Enquanto o paradigma agroextrativista está relacionado à ideia de uma natureza viva, na qual a floresta é meio de produção, o paradigma mecânico-químico-genético está ligado à ideia de uma natureza morta, em que a floresta é insumo, um recurso a ser consumido.

Costa ^9^ ^,^ ^10^ identificou seis TTs presentes na economia agrária regional, sendo três associadas aos agentes camponeses e três aos patronais. O Quadro 1 mostra uma síntese dessas TTs e descreve os padrões e arranjos que podem ser observados para os elementos presentes na paisagem, moldada por esses agentes e seus sistemas técnicos produtivos.

**Quadro 1:** Descrição das trajetórias tecnológicas (TTs) em associação com elementos presentes na paisagem modificada.

TTS	AGENTE	CARACTERIZAÇÃO DAS TTS	PAISAGEM
TT1 Sistema de cultivo (agricultura temporária)	Camponês	Com base em um *paradigma agropecuário*, essa TT está associada a um sistema diversificado de culturas temporárias, como mandioca, abacaxi, milho e arroz. Podemos caracterizá-lo como uma trajetória tecno-produtiva camponesa diversificada e relativamente especializada, na qual a produção agrícola é predominantemente conduzida por unidades familiares	Paisagem diversificada, incluindo combinações de agricultura de pequena escala temporária, diferentes estádios de vegetação secundária, áreas reduzidas de pastagens e extensões contínuas de florestas
TT2 Agroflorestal		Com base em um *paradigma agroextrativista*, essa TT adota uma abordagem diversificada no uso dos recursos naturais, com um pressuposto de preservação dos recursos naturais associados ao Bioma Amazônico. A atividade predominante é o extrativismo não-madeireiro, que se baseia em sistemas agroflorestais (SAFs), podendo ou não ser intercalada com pequenas áreas destinadas à agricultura permanente, silvicultura e culturas temporárias. A produção rural é predominantemente conduzida por unidades familiares	Paisagem caracterizada pela predominância de floresta primária, podendo ocorrer manchas de vegetação secundária, agricultura de pequena escala e pequenas áreas de pastagem
TT3 Pecuária de corte		Com base em um *paradigma agropecuário*, essa TT se destaca pelo uso extensivo da terra, com a pecuária de corte como sua atividade central, possivelmente acompanhada pela pecuária leiteira e pela agricultura temporária. Esse sistema baseia-se, principalmente, na mão de obra familiar, com nível de especialização relativamente baixo em comparação à TT4	Paisagem caracterizada por pastagens de pequeno e médio porte, algumas áreas com arbustos. Áreas reduzidas de agricultura de pequena escala, podendo conter áreas com vegetação, em estágios iniciais, e fragmentos de floresta
TT4 Pecuária de corte	Patronal	Com base em um *paradigma agropecuário*, essa atividade se apoia no uso extensivo da terra para a criação de gado para corte, apresentando um grau maior de especialização em comparação com a TT3. Sua execução envolve mão de obra assalariada, no entanto, com uma taxa de emprego relativamente reduzida	Paisagem homogênea, caracterizada por áreas extensas e uniformes de pastagens manejadas, podendo ocorrer fragmentos isolados de floresta primária e secundária
TT5&6 Agricultura permanente (silvicultura)		Com base em um *paradigma agropecuário*, a TT5 se refere à monocultura empresarial especializada na agricultura permanente e adota uma abordagem intensiva no uso do solo. Por outro lado, a TT6 tem sua base na silvicultura. Ambas as TTs dependem de mão de obra assalariada e da aplicação de métodos *mecânico-químico-genético*	Paisagem homogênea formada pelo cultivo de um número limitado de espécies arbóreas e arbustivas, podendo ocorrer fragmentos isolados de floresta primária. No caso de floresta manejada, pode ocorrer a extração seletiva de madeira
TT7 Agricultura anual		Com base em um *paradigma agropecuário*, essa TT baseia-se na agricultura temporária, especializando-se, principalmente, na produção de grãos como soja e milho. Faz uso intensivo do solo, incorporando tecnologias *mecânico-químico-genético* e demanda a utilização de mão de obra assalariada	Paisagem homogênea, caracterizada por extensas e homogêneas áreas de cultivo agrícola, podendo ocorrer ou não fragmentos isolados de floresta primária

Fonte: adaptado de Costa ^4^ ^,^ ^9^ e de Codeço et al. ^11^.

### Identificando as PLU

A primeira etapa metodológica consiste na escolha da resolução da célula. Cada célula representa uma unidade de paisagem, e o seu conjunto forma uma grade (espaço celular) ^7^. As informações relativas ao uso e cobertura da terra, derivadas de imagens satélites, em geral, são fornecidas de forma sistemática, com resolução espacial média que varia de 10m, 20m ou 30m. Assim, para avaliar o arranjo espacial e a composição das classes de uso e cobertura da terra que compõem as paisagens de produção, trabalhamos com uma grade de células, com resolução espacial de [2×2]km. A definição do tamanho da célula é feita de forma empírica ^7^ e envolve o uso de imagens de satélite, de média resolução, dados de uso de cobertura da terra e registros de campo, integrados e analisados em um Sistema de Informação Geográfica (SIG). A definição do tamanho das células depende das características da região, dos objetos e categorias de interesse ^7^. Desse modo, considerou-se o tamanho das áreas de uso agropecuário que variam na área de estudo, entre 0,5 e 2ha para camponeses e acima desse valor para patronais médios e grandes, podendo chegar a 200ha ou mais. Com base no tamanho de células definido, foram geradas para a área de estudo 1.291 células: 290 para o Município de Mocajuba e 1.001 para Cametá.

A construção das PLU é influenciada pelo tamanho das células que irão delimitar os padrões das paisagens de interesse. A descrição delas é feita observando nas bases de dados os arranjos espaciais e a composição dos elementos de paisagem (polígonos e classes de uso e cobertura da terra) presentes na célula, além das informações de contexto, como densidade e tamanho de estabelecimentos rurais, proximidade com rios, localização em ilha ou áreas de várzea. Para essa construção, partimos do princípio de que os sistemas técnicos de produção rural apresentam paisagens com padrões espaciais específicos e visualmente identificáveis nos dados derivados de imagem de satélite, apresentando correspondências com as TTs, mas não necessariamente uma relação direta.

Para a construção da tipologia das PLU para a região é necessário considerar seu contexto econômico e agrário. Os municípios de Cametá e Mocajuba, alvo deste estudo, inserem-se na região do Baixo Tocantins, no Pará, segunda maior região produtora de açaí do Pará ^21^. Essa região apresenta significativa produção agroextrativista e agrícola, baseada em sistemas técnicos camponeses. O açaí é produzido em diferentes sistemas, em áreas de ilhas, várzeas e, mais recentemente, em áreas de terra firme ^22^. Também com uma economia expressiva, temos a agricultura de pequena escala, localizada em áreas de terra firme, associadas ao cultivo de pimenta-do-reino, cacau e mandioca, esta última produzida em sistema de pousio ^22^ ^,^ ^23^. Junto aos camponeses e seus sistemas técnicos, coexistem os agentes patronais e seus modos de produzir, em particular, associados à produção do açaí de terra firme. Além do açaí produzido em sistemas irrigados e mecanizados, estão presentes a produção de dendê, pimenta-do-reino e cacau, estes dois últimos frequentemente consorciados com o açaí ^22^. Observa-se ainda a piscicultura, desenvolvida junto à produção agrícola, em sistemas patronais e camponeses. Importante destacar que a área, ao contrário da maioria das regiões da Amazônia, apresenta pouca atividade de pecuária ^22^ ^,^ ^24^.

Visando construir uma tipologia representativa das categorias de PLU presentes na região para o ano de 2021, uma importante informação foi incorporada aos dados de uso e cobertura da terra, o indicador de áreas potenciais de ocorrência de palmeiras nativas de açaí. Dada a natureza diversa dos sistemas produtivos do açaí, os dados de produção sistemática disponíveis de uso e cobertura da terra, como os do TerraClass ^14^ e MapBiomas (https://brasil.mapbiomas.org/) ^25^, não foram utilizados, pois não identificam a agricultura de pequena escala, nem discriminam estágios da vegetação secundária, associados em grande parte aos cultivos em sistema de pousio. A necessidade de uma melhor identificação dos cultivos levou à realização de uma classificação utilizando imagens de satélites do Sentinel-2A (https://www.engesat.com.br/sentinel-2/), com 10m de resolução espacial, para a obtenção de classes de uso e cobertura mais detalhadas e aderentes às características da área de estudo.

Para a classificação, foram utilizados cubos de dados de imagens do Sentinel-2A ^26^ e classificação supervisionada utilizando o Random Forest ^27^, implementado no pacote SITS (*Satellite Image Time Series Analysis on Earth Observation Data Cubes*) ^26^. O cubo foi produzido com imagens mensais do período de 01/Jan/2021 a 31/Dez/2021. Foram utilizadas dez bandas espectrais, exceto a banda de vapor d’água. Foi gerado um mapa de uso e cobertura da terra, que apresentou exatidão global de 94% e coeficiente kappa de 0,92, contendo as seguintes classes: (1) agricultura de pequena escala, (2) agricultura de larga escala, (3) pasto limpo, (4) pasto sujo, (5) vegetação secundária inicial, (6) vegetação secundária avançada e (7) hidrografia. O mapa final inclui as classes floresta e não-floresta, provenientes do PRODES ^13^.

O indicador de áreas potenciais de ocorrência de palmeiras nativas de açaí é elaborado a partir da combinação entre dados de cobertura florestal e dados dos ambientes hidro-topográficos ^28^. Um mapa hidro-topográfico é gerado para indicar áreas de baixio, zonas de transição (entre várzeas e vertentes), áreas de vertentes e áreas de platô (terra firme). Os dados gerados pelo mapeamento hidro-topográfico foram combinados com os dados de cobertura florestal. Desta forma, as áreas com alto potencial para ocorrência de açaí nativo (*Euterpe oleracea*) foram definidas por apresentarem cobertura florestal primária ou secundária, em áreas de várzea (baixo) ou em zonas de transição. As áreas com moderado potencial foram identificadas em locais com cobertura florestal em áreas de vertente, enquanto áreas de baixo potencial foram identificadas por apresentarem vegetação secundária e/ou floresta em terra firme ^24^. As áreas que não apresentaram floresta primária ou vegetação secundária foram definidas como áreas de ausência de palmeiras de açaí. A classe de alto potencial de ocorrência de palmeiras de açaí foi integrada aos dados de uso e cobertura da terra, formando uma nova base unificada. Essa integração é importante na determinação dos tipos de PLU presentes na tipologia construída.

A base unificada de uso e cobertura da terra foi agregada às células de [2×2]km, possibilitando definir cinco (5) tipos de PLU presentes na área de estudo. Três PLU associadas aos sistemas camponeses e duas aos sistemas patronais, sendo elas: (1) unidade camponesa extrativista - PLU1; (2) unidade camponesa agroextrativista com agricultura temporária - PLU2; (3) unidade camponesa com produção de açaí, agricultura permanente e/ou temporária e pastagens - PLU3; (4) unidade patronal com açaí plantado irrigado - PLU4; e (5) unidade patronal com pecuária de corte - PLU5. A Figura 1 ilustra os padrões espaciais relativos ao novo mapa de uso e cobertura da terra que caracterizam os tipos de PLU identificados na área de estudo.

**Figura 1:** Padrões das unidades de paisagem da produção (PLU, acrônimo em inglês) observados com o dado de uso e cobertura da terra utilizando células de [2×2]km.

PLU1: unidade camponesa extrativista; PLU2: unidade camponesa agroextrativista com agricultura temporária; PLU3: unidade camponesa com produção de açaí, agricultura permanente e/ou temporária e pastagens; PLU4: unidade patronal com açaí plantado irrigado; PLU5: unidade patronal com pecuária de corte.

As PLU caracterizadas neste estudo foram desenvolvidas considerando que os sistemas técnicos associados à produção do açaí são predominantes na região. No entanto, em ambientes de terra firme, pode-se encontrar outros tipos de cultivos, como a pimenta-do-reino e o dendê, que podem ou não incluir o açaí, além das áreas de pecuária que não incluem açaí. Como as PLU se encontram inseridas em diferentes contextos de paisagens, por exemplo os ambientes de ilhas, várzea e terra firme, os cultivos e as práticas tecnológicas adotadas no manejo, colheita e armazenamento da produção são diferenciados. Consequentemente, também são diferenciadas as relações dessas PLU com fatores de risco/proteção no contexto da saúde local. A tipologia final, abrangendo os cinco tipos de PLU, é apresentada no Quadro 2.

**Quadro 2:** Tipologia das unidades de paisagem da produção (PLU, acrônimo em inglês) associadas aos diferentes sistemas técnicos de produção agropecuária e sua correspondência com as categorias econômicas analíticas descritas pelas trajetórias tecnológicas (TTs).

TIPO	ESTRUTURA, COMPOSIÇÃO E CONTEXTO DA PAISAGEM	CARACTERIZAÇÃO DAS PLU	TT
PLU1 Unidade camponesa extrativista	Áreas com predominância de classes de *alto potencial de ocorrência de palmeiras de açaí e manchas de floresta*, podendo conter manchas isoladas de *vegetação secundária avançada* **Contexto**: região de ilhas ou próximas ao rio Tocantis. Células com alta densidade de estabelecimentos rurais, sendo que os lotes não ultrapassam 280ha	Sistema baseado em um *paradigma agroextrativista*. Essa PLU se caracteriza por um *Sistema Agroflorestal* (SAFs-F) originário ao bioma. Nessa unidade, o extrativismo do açaí se destaca como atividade econômica principal, podendo ser complementado pela pesca. Como um produto secundário, pode-se encontrar cacau plantado próximo às áreas de açaí. Predomina a mão de obra familiar (mais de 50%), complementada por trabalhadores diaristas durante o período de colheita de açaí	TT2
PLU2 Unidade camponesa agroextrativista com agricultura temporária	Paisagem diversificada. Áreas com predominância de classes de *alto potencial de ocorrência de palmeiras de açaí* e floresta, incluindo pequenos talhões de agricultura de pequena escala, vegetação secundária avançada e pequenos fragmentos de *vegetação secundária inicial* **Contexto:** região de várzea do rio Tocantins e margens de igarapés. Células com alta densidade de estabelecimentos rurais, sendo que os lotes não ultrapassam 280ha	Baseada em um *paradigma agroextrativista*, essa PLU se caracteriza por um *Sistema Agroflorestal* (SAFs-A), combina o manejo florestal, especialmente do açaó, com práticas agrícolas. Nessa unidade, a economia do açaí é complementada por agricultura temporária, realizada em áreas não afetadas pela inundação sazonal dos rios, como o cultivo da mandioca, em sistemas de roçado com o uso de sistemas de pousio. Como um produto secundário, pode-se encontrar o cacau, plantado próximo às áreas de açaí ou em terra firme, bem como a pimenta-do-reino. A mão de obra é predominantemente familiar e pode ser complementada por trabalhadores diaristas durante o período de coleta do açaí	TT2
PLU3 Unidade camponesa com produção de açaí, agricultura permanente e/ou temporária e pastagens	Paisagem diversificada. Áreas caracterizadas por um mosaico de classes com a*lto potencial de ocorrência de palmeiras de açaí, agricultura em pequena escala, manchas de vegetação secundária inicial*, podendo incluir pequenas áreas isoladas de pasto limpo e sujo com forma irregular **Contexto:** região de terra firme. Estão mais distantes das várzeas e ilhas do rio Tocantins. Células com moderada densidade de estabelecimentos rurais, sendo que os lotes não ultrapassam 280ha	Baseando-se em um *paradigma agropecuário*, essa PLU se destaca por apresentar um sistema diversificado de culturas temporárias e permanentes com alguma especialização. O açaí, junto à produção de pimenta-do-reino e mandioca, constitui uma das atividades relevantes, sem ser necessariamente a principal atividade, em termos de área e valor de produção. A produção do açaí pode ocorrer em consórcio com um conjunto de culturas permanentes, como a pimenta-do-reino e o cacau. O cultivo de mandioca em sistemas de pousio e a produção de farinha são atividades importantes tanto para o consumo quanto para a renda das famílias. Também pode apresentar tanques de piscicultura. A produção é majoritariamente conduzida por mão de obra familiar	TT2
PLU4 Unidade patronal com açaí plantado irrigado	Paisagem homogênea. Áreas com predominância da classe *agricultura de larga escala* com forma geométrica regular. Podem apresentar manchas isoladas de *vegetação secundária em diferentes estágios* e áreas de pastagem com formas geométricas bem definidas **Contexto:** região de terra firme, distante do rio Tocantins. Células com baixa densidade de estabelecimentos rurais, sendo que os lotes têm tamanho superior a 280ha	Baseando-se em um *paradigma agropecuário*, essa PLU se destaca pelo uso mais intensivo da terra, adotando um sistema especializado na agricultura empresarial, apoiado no modelo *mecânico-químico-genético*. Embora tenha suas bases na produção do açaí, também pode integrar outras culturas, como pimenta-do-reino, cacau e cupuaçu. A pimenta-do-reino e o cacau podem ser cultivados em consórcio com o açaí. Pode ocorrer ou não dendê e piscicultura. A produção é majoritariamente conduzida por mão de obra assalariada	TT5&6
PLU5 Unidade patronal com pecuária de corte	Paisagem homogênea. Áreas com predominância das classes *pasto limpo e pasto sujo* com forma geométrica bem definida. Podendo ocorrer fragmentos isolados de *floresta ou vegetação secundária avançada* **Contexto:** região de terra firme, distante do rio Tocantins. Células com baixa densidade de estabelecimentos rurais, sendo que os lotes têm tamanho superior a 280ha	Baseada em um *paradigma agropecuário*, essa PLU é caracterizada pelo uso extensivo da terra em um sistema empresarial voltado para a criação de gado, predominantemente conduzido por mão de obra assalariada	TT4

### Mapeando as PLU

A classificação das PLU foi realizada tendo como base a tipologia desenvolvida (Quadro 2). Atributos espaciais, composição e contexto são extraídos para cada célula de [2×2]km, a fim de classificar as PLU; nesse processo, utilizamos um classificador supervisionado baseado em árvores de decisão com método *boosting* ^18^. Os atributos, ou descritores de paisagem, são utilizados para treinar os algoritmos que irão realizar a classificação das PLU de maneira supervisionada. O processo de coleta de amostras é realizado de forma iterativa até que se obtenha um conjunto consistente de amostras de treinamento que represente adequadamente a tipologia elaborada. O método *boosting* é utilizado para melhorar a acurácia da classificação criando múltiplas árvores de decisão com base em um número predefinido de iterações, em que, a cada iteração, é realizado o ajuste dos erros de treinamento ocorridos na iteração anterior ^18^. Combinando várias árvores, com diferentes métricas de composição, estrutura e contexto da paisagem, a decisão final do modelo resulta de um sistema de votação que atribui à cada célula a classe mais frequente imputada pelas árvores de decisão ^7^ ^,^ ^18^.

A classificação das PLU consistiu em quatro etapas e foi realizada dentro do ambiente integrado *Geographic Data Mining Analyst-GeoDMA* ^29^:

i) Extração de atributos. Utilizando os dados de uso e cobertura da terra, foram extraídas 246 métricas de composição e estrutura da paisagem e de contexto, como distância a rios, distância a estradas, presença/ausência de rios, número e tamanho dos estabelecimentos rurais para cada célula;

ii) Seleção de amostras de treinamento e teste. Foram coletadas 212 amostras: 48 amostras para as PLU 1, 2, 3 e 5; e 20 amostras para a PLU4. O número inferior de amostras para a PLU4 ocorreu devido à menor representatividade de células com o padrão espacial correspondente. Entre as 212 amostras coletadas, 70% foram destinadas ao treinamento do classificador e 30% foram utilizadas na fase de validação como amostras de teste;

iii) Classificação das células por meio de algoritmo de árvore de decisão com *boosting*, com 99 iterações e classificações independentes.

iv) Avaliação da classificação. A avaliação foi realizada por meio de matriz de confusão com o conjunto de amostras de teste.

## Resultados

A Figura 2a apresenta o resultado do mapeamento das PLU, incluindo uma área ampliada. As PLU camponesas (PLU1, PLU2, PLU3) cobrem 95% das células, evidenciando a dominância das atividades econômicas desses agentes na região estudada (Figura 2b). A PLU2 representa mais de 45% (496 células) do total das células mapeadas, seguida pela PLU1, que corresponde a quase 31% (335 células), e pela PLU3 com 18% (202 células). A PLU4 corresponde a 4,1% (44 células), enquanto a PLU5 não alcança 1% (8 células). Em alinhamento com as TTs, as PLU1 e PLU2 estão associadas à TT2, representando juntas quase 77% do total mapeado na região, o que corrobora os estudos de Nogueira et al. ^23^, e com registros de campo ^22^ que indicam a predominância de atividades de agentes camponeses na região. Considerando o último censo agropecuário (2017), a trajetória camponesa TT2 é dominante nos dois municípios. Quanto à trajetória patronal, prevalece em Cametá a TT5&6 e em Mocajuba a TT7 ^12^.

**Figura 2:** Resultados do mapeamento e área ampliada com detalhes das unidades de paisagem da produção (PLU, acrônimo em inglês), e número de células e as porcentagens correspondentes a cada PLU presente na área de estudo. Amazônia brasileira, 2021.

PLU1: unidade camponesa extrativista; PLU2: unidade camponesa agroextrativista com agricultura temporária; PLU3: unidade camponesa com produção de açaí, agricultura permanente e/ou temporária e pastagens; PLU4: unidade patronal com açaí plantado irrigado; PLU5: unidade patronal com pecuária de corte.

Na classificação supervisionada ^18^, foram geradas seis árvores de decisão para classificar as PLU. A parametrização do algoritmo previa 99 iterações, contudo, foi estabilizado no sexto ciclo de treinamento. O erro de ajuste do modelo variou entre 0,6% e 4,4% até se estabilizar na última iteração em 0%. A escolha dos atributos é realizada por meio de uma medida de entropia que estima o ganho de informação das variáveis durante a classificação, descartando aquelas com menores ganhos. Os atributos selecionados são apresentados na Figura 3a. Das 15 métricas usadas nas árvores de decisão, quatro foram usadas na raiz, gerando os ramos iniciais: (1) área da vegetação secundária avançada (CA_VSA); (2) índice de forma da classe pasto limpo (LSI_PL); (3) densidade de manchas das áreas de agricultura de larga escala (PD_AGLE); e (4) índice de dimensão fractal das áreas de agricultura de larga escala (AWMPFD_AGLE). A maioria das métricas foi utilizada para diferenciar as PLU4 ou PLU5 das demais, com exceção da CA_VSA, que separou a PLU1 no primeiro corte. Nos ramos intermediários e finais das árvores, outras métricas se destacaram, como tamanho médio da classe agricultura de larga escala (MPS_AGPE), além de três métricas de contexto relativas à distância mínima (HID_min) e presença de rios (P_HID) e IJI_HID (índice de interspersão e justaposição da hidrografia), usadas para separar a PLU2 da PLU3. A PLU2 localiza-se próxima aos rios, onde predominam atividades como manejo e coleta de açaí, com áreas menores dedicadas à agricultura; enquanto a PLU3, situada mais distante dos rios, tem a agricultura como atividade econômica principal, podendo, ou não, incluir cultivos de açaí.

**Figura 3:** Descrição das 15 métricas utilizadas na classificação das unidades de paisagem da produção (PLU, acrônimo em inglês), árvore de decisão e matriz de confusão para as PLU classificadas usando amostras de teste.

PLU1: unidade camponesa extrativista; PLU2: unidade camponesa agroextrativista com agricultura temporária; PLU3: unidade camponesa com produção de açaí, agricultura permanente e/ou temporária e pastagens; PLU4: unidade patronal com açaí plantado irrigado; PLU5: unidade patronal com pecuária de corte.

Em uma das árvores, a métrica TABO_alto, que mede a área do maior objeto da classe alto potencial de ocorrência de palmeiras de açaí, foi utilizada. Essa métrica separou PLU3 de PLU2. Esse resultado mostra a importância da diferenciação das classes de agricultura no dado de entrada para a distinção das PLU patronais e camponesas. Nenhuma métrica de composição da paisagem, densidade e tamanho de estabelecimentos agropecuários foi utilizada. Melhorias e atualizações das bases de dados de entrada poderiam melhorar, por exemplo, a separação entre unidades camponesas e unidades patronais. Na Figura 3b, é apresentada uma das seis árvores geradas com as métricas de estrutura de paisagem e de contexto.

A matriz de confusão da Figura 3c possibilita uma avaliação da classificação com 30% das amostras de teste. Houve poucas confusões, a mais significativa aconteceu entre a PLU3, camponesa, e a PLU5, patronal associada à pecuária. Isso ocorreu, porque tanto a PLU3 quanto a PLU5, localizadas em terra firme, podem apresentar classes como pasto sujo, vegetação secundária inicial e floresta. O desbalanceamento nas amostras de treinamento é uma limitação desse método. A PLU1 foi confundida com a PLU2 em 7% das células. Não houve confusões entre PLU2 e PLU4, embora a PLU4 apresente um número limitado de amostras.

## Discussão

Os resultados da classificação são consistentes com as expectativas para essa região, alinhando-se aos registros de campo ^22^ e trabalhos que incorporam as TTs ^11^ ^,^ ^23^. O mapeamento das PLU em escala intramunicipal mostrou, em 2021, a persistência das TTs camponesas associadas às PLU1, PLU2 e PLU3. As PLU1 e PLU2 predominam nas ilhas e várzeas do baixo Tocantins e de seus afluentes. À medida que se distancia das áreas alagáveis, identifica-se a presença da PLU3. As PLU4 e PLU5, associadas às TTs patronais, embora menos expressivas, apresentam-se mais afastadas do baixo Tocantins, localizando-se próximas das estradas e rodovias, em áreas de terra firme, em que atividades como pecuária e monocultura do açaí são desenvolvidas, configurando padrões de paisagens homogêneos. As PLU, associadas às TTs, coexistem e interagem de maneiras distintas à dinâmica econômica do rural amazônico. As alterações na estrutura e composição das paisagens florestais dos modos de viver e produzir, associados aos sistemas técnicos, são capturadas pelas PLU. Desta forma, as PLU se constituem em uma estrutura de representação que introduz novas possibilidades para (re)pensar os elementos de exposição e contato das populações nessas áreas a velhos e novos patógenos ^30^ e as abordagens integradas saúde-ambiente-economia.

Doenças como leishmaniose visceral (LV), leishmaniose cutânea (LC), malária, dengue, doença de Chagas, por exemplo, são doenças que prevalecem na Amazônia até os dias atuais ^11^ ^,^ ^31^. A base de dados do *Trajetórias* ^12^, construída para todos os municípios da Amazônia Legal, apresenta as taxas de incidência das principais doenças presentes na área estudada, estratificadas pelo meio urbano e rural. Na base de dados do projeto *Trajetórias* ^12^, em Cametá, no período de 2015 a 2019, observa-se que a taxa de incidência de malária (*Plasmodium vivax*) no ambiente rural, considerando a taxa por 100 mil habitantes, foi de 4.620,19. A doença de Chagas teve uma taxa de 22,61, enquanto LV foi de 27,60 e LC de 6,65. Em Mocajuba, não foram registrados casos de doença de Chagas entre 2015 e 2019 no ambiente rural. A taxa de incidência de malária (*Plasmodium vivax*) foi de 2.466,58, a LV foi de 31,54 e a LC foi de 40,15.

As paisagens da produção PLU1 e PLU2 apresentam condições socioambientais que favorecem a ocorrência de malária (*Plasmodium vivax*), doença de Chagas e LC, na área de estudo. Em comunidades com produção de açaí no baixo Tocantins, foram registrados casos de doença de Chagas com transmissão por via oral ^32^. Em geral, é nas áreas de ilha e várzea que o açaí de produção camponesa ocorre, representado pelas PLU1 e PLU2. Os processos de colheita do fruto, higienização e armazenamento introduzem riscos para transmissão via oral, uma vez que os frutos podem ser contaminados com fezes e urina do vetor, contendo o *Trypanosoma cruzi* ^33^, em qualquer uma destas fases. Embora sejam dadas orientações aos camponeses por agentes das instituições locais e pelas indústrias que compram o fruto do açaí, na prática elas são ainda insuficientes para o controle efetivo da doença, resultando na incidência de doença de Chagas na região. No caso da LC, o estudo de Sousa Júnior et al. ^31^ mostra que, entre 2007 e 2016, áreas de ilhas e várzeas em Cametá tiveram os maiores números de casos de LC.

A leishmaniose é transmitida, predominantemente, pela picada de flebotomíneos (*birigüi*, mosquito palha) do gênero *Lutzomyia*. Esses mosquitos utilizam locais úmidos, com sombra e matéria orgânica, como folhas e frutos, além de fezes de animais, para se reproduzirem. Tocas de animais, base ou tronco de árvores são potenciais criadouros. Alimentam-se de sangue de diversas espécies de animais silvestres, animais sinantrópicos e, em ciclos específicos de transmissão, de animais domésticos ^31^. Estes podem ser hospedeiros naturais ou (potenciais) reservatórios silvestres. Nas PLU1 e PLU2, temos situações de casas muito próximas à floresta e regiões de mata, estas próximas ao ambiente domiciliar e das áreas de produção e, ainda, há presença de animais domésticos e silvestres, em particular, roedores. São paisagens que aumentam os riscos de exposição e contato dos flebotomíneos com produtores camponeses. Orientações relativas ao manejo dos territórios de produção, que no caso do camponês envolve seu domicílio e peridomicílio, como limpeza de quintais e de suas áreas de cultivo e extração, pode ser uma estratégia desenhada pela vigilância em saúde, integrada com outras políticas municipais.

A PLU3, embora camponesa, tem um sistema técnico diferenciado. Localiza-se em paisagens mais diversas, com produção de açaí, agricultura permanente e/ou temporária e pastagens. Apresenta condições socioambientais que favorecem o risco de transmissão para LC e LV. Ocorre na terra firme, abrangendo as franjas urbanas e distante dos rios, mas está próxima de estradas vicinais. Encontra-se em regiões onde os distúrbios do ecossistema florestal são mais acentuados do que na PLU1 e PLU2. O desmatamento, proximidade de estradas vicinais e proximidade a áreas urbanas adensadas, características dessas PLU, aumentam os riscos para leishmaniose (cutânea e visceral), como observado no baixo Tocantins ^31^ ^,^ ^34^. No caso destas, as questões relativas aos processos da cadeia de produção do açaí, coleta, higienização e armazenamento, associadas à doença de Chagas, também estão presentes ^33^.

A PLU4, patronal, está associada à monocultura do açaí. Esta tem um pacote de solução tecnológica com emprego de mecânica, química e genética, contando com assistência técnica especializada. Porém, produz um ambiente pouco diversificado, com pouca cobertura florestal. A circulação da fauna silvestre se restringe aos fragmentos de mata próximos e à nova “floresta” de palmeiras plantadas, em geral, consorciada com outra cultura. Este ambiente favorece a transmissão de doenças que têm em seu ciclo animais silvestres, que são hospedeiros e reservatórios de uma ampla gama de espécies parasitárias. O açaí em consórcio com o cacau apresenta condições socioambientais favoráveis à contaminação por LV ^34^. Por outro lado, a densidade de população nessas áreas é menor, uma vez que emprega pouca mão de obra, reduzindo as chances de contato e riscos de transmissão no local ou em áreas vizinhas.

A PLU5, patronal, é caracterizada pela prática de pecuária extensiva. Os sistemas técnicos da pecuária extensiva consomem significativamente a cobertura florestal que é substituída por pastagem. Desta forma, desenham zonas de transição entre áreas de pasto e áreas de mata, que se tornam habitats propícios para os roedores silvestres ^35^. As paisagens da produção da pecuária em Cametá e próximas das margens do rio Tocantins registraram ocorrências de LC ^31^. Para esses territórios, dos tipos PLU4 e PLU5, a vigilância em saúde pode desenhar ações específicas e estabelecer, em conjunto com outros setores públicos municipais e com os agentes patronais, procedimentos para monitorar e controlar a presença e circulação de animais silvestres, no interesse da saúde pública local.

Nesta análise, ainda inicial e de teor descritivo, observamos como identificar, caracterizar e mapear as PLU, ajudando a compreender os riscos diferenciados de exposição humana a agentes patogênicos. Um quadro analítico que integra elementos socioambientais no contexto da produção agrária se mostra uma estrutura promissora a ser investigada. Embora as PLU tenham sido mapeadas para apenas um ano, em região específica, elas podem ser produzidas para vários anos e adaptadas para outras regiões, permitindo observar a dinâmica dos sistemas técnicos e das TTs ^7^, sua evolução, e em quais direções suas dinâmicas alteram as paisagens do bioma, na escala intramunicipal. Dados espaciais derivados de imagens de satélite possibilitam gerar análises refinadas em diferentes escalas espaciais e temporais ^7^ ^,^ ^24^.

Não exploramos, neste artigo, a PLU como variável em modelos estatísticos ou mistos para estudar as relações entre suas dinâmicas e a emergência, reemergência ou intensificação de processos saúde-doença. Nosso foco foi apontar essa nova estrutura integradora como uma representação territorial da tríade saúde-ambiente-economia e seus potenciais em estudos integrados em saúde. Compreender a interação entre os agentes sociais que promovem as economias no bioma, as paisagens que eles transformam e seus impactos nos processos saúde-doença é essencial para desenvolver (novas) estratégias de vigilância e controle ou repensar as existentes. Neste processo, deve-se considerar a constante transformação do ecossistema florestal, dirigida pelas decisões de seus agentes econômicos e seus impactos na saúde pública ^11^ ^,^ ^16^ sobre as comunidades e todas as populações amazônidas.

A estrutura apresentada pode ajudar a instrumentalizar os debates na dimensão saúde, sobre políticas e propostas de estratégias mais eficazes, associadas com escolhas de caminhos para o desenvolvimento econômico agrário na Amazônia brasileira. No momento, está em curso uma disputa sobre projetos de Bioeconomia ^20^ como estratégia para o desenvolvimento regional. Entender sobre que Bioeconomia estamos falando e, em particular, como nossas opções por um projeto ou outro impactam a saúde do ecossistema florestal e a saúde das populações na Amazônia é necessário e urgente.
